# Prioritising child health and maternity evidence-based interventions or service models: a stakeholder-driven process

**DOI:** 10.1186/s12913-022-08110-2

**Published:** 2022-06-10

**Authors:** Camilla Forbes, Naomi Morley, Kristin Liabo, Gretchen Bjornstad, Heather Boult, Shafiq Ahmed, Kayley Ciesla, Yassaman Vafai, Sally Bridges, Stuart Logan, Vashti Berry

**Affiliations:** 1 University of Exeter, College of Medicine and Health, South Cloisters, Exeter, EX1 2LU UK; 2 PenARC, Exeter, UK; 3 Bradford Teaching Hospitals NHS Foundation Trust, Bradford, UK; 4 University of York, Heslington, York, YO10 5DD UK

**Keywords:** Priority-setting, Stakeholder involvement, APEASE criteria

## Abstract

**Aim:**

A UK programme, led by the National Institute for Health Research (NIHR) (https://www.nihr.ac.uk) and coordinated by Applied Research Collaborations (ARC), (https://www.nihr.ac.uk/explore-nihr/support/collaborating-in-applied-health-research.htm) aimed to identify and select evidence-based, implementation-ready service innovations for evaluation. The programme focused on seven areas of health provision. We report on a prioritisation process designed to identify and assess innovations in one of these areas: child and maternal health (CH&M).

**Methods:**

We developed a three-stage, online, stakeholder driven process to 1) identify, 2) assess and prioritise and 3) select evidence-based interventions or service models, using crowdsourcing to identify projects and the APEASE criteria to assess and select projects. A brief evidence review was conducted for all initial suggestions to identify those with the largest evidence-base to take forward for ranking by stakeholders. Stakeholder workshops considered and ranked these suggestions using the APEASE criteria. We then conducted in-depth evidence reviews for the highest ranked suggestions. The Project Management Group and Advisory Board used these reviews and the APEASE criteria to select the final projects.

**Results:**

We received 32 initial suggestions from a range of clinicians, practitioners and researchers. Fourteen of the most evidence-based suggestions were considered and ranked at four themed stakeholder workshops. Nine suggestions were ranked for further in-depth evidence review and a final four projects were selected for implementation evaluation using the APEASE criteria. These were: 1. Maternal Mental Health Services Multidisciplinary Teams 2. Early years tooth brushing programme 3. Trauma-focused CBT for young people in care and 4. Independent Domestic Violence Advisors in maternity settings. Feedback from participants suggested that having public representatives participating in all stakeholder meetings, rather than being consulted separately, focused discussions clearly on patient benefit rather than research aims.

**Conclusions:**

The stakeholder-driven process achieved its aim of identifying, prioritising and assessing and selecting, evidence-based projects for wider implementation and evaluation. The concurrent process could be adapted by other researchers or policy makers.

**Supplementary Information:**

The online version contains supplementary material available at 10.1186/s12913-022-08110-2.

## Background

Priority setting in healthcare, the process of making decisions about how best to allocate resources to improve population health, is a necessity as all healthcare systems have limited amounts of resources. In the UK and elsewhere, there has been an increased interest in bringing in different perspectives, including patients, health care professionals and members of the public into decision-making about which healthcare services to prioritise [1]. People with ‘lived’ or ‘professional’ expertise of health care services are considered to bring a particular type of knowledge to the prioritisation process, which would be missed if the process is informed by researchers and managers only. This is thought to improve service uptake and engagement further down the line, increase equity, and contribute to a more transparent process of prioritisation [1]. When working with a range of stakeholders it is important to find a process that feels inclusive and accessible to the different stakeholders involved. Without this, there is a risk of alienating stakeholders, which in turn might be detrimental to service engagement and uptake.

Different approaches and criteria suit different circumstances and decision-makers need to ensure that they select the best process and criteria for their specific context and remit [2–4]. Methods used in priority setting processes are varied, and include surveys, Delphi studies, one-day events, workshops or focus groups [5–7]. Each offers advantages and disadvantages and reflection is needed for the best method for a given prioritisation remit; for example Delphi studies can reach a larger number of stakeholders compared to workshops or focus groups but can also limit the pool of particular groups and may not operate at the same level of detail/depth as in-person events [8]. Lavallee et al. compared three approaches (Delphi survey, on-line crowd-voting and in-person focus groups) and reported that the focus group participants evaluated their experience the highest [5]. We found a limited number of approaches in priority setting health interventions for child and maternal health [9, 10], each with a distinct concern. For example: the Child Health and Nutrition Research Initiative (CHNRI) [11], aims to inform those who invest in research about the risks associated with their investments and the James Lind Alliance (JLA) Priority Setting Partnerships [12], aims to identify areas where there are unanswered questions about treatments. Approaches use different multi or single criteria to assess initiatives depending on their particular remit, such as multi-criteria decision analysis (MCDA) [3], as opposed to single criteria, such as cost-effectiveness analysis. Multi criteria such as MCDA have been advocated [13], although found to have important challenges when used to assess patient preference [14]. APEASE (Table 1), designed by Mitchie et al., offers a multi-criterion tool that was developed for the design and evaluation of interventions [15, 16]. It has been utilised in numerous ways to design and evaluate interventions however, it has rarely been used in priority setting [17]. APEASE has a simple set of six criterion, which is accessible for stakeholders with differing knowledge and experience. In addition, the APEASE criteria is flexible to different priority setting methodologies although to our knowledge, its only use to date has been in a survey capacity [17].

**Table 1:** The APEASE criteria for designing and evaluating interventions (replicated with permission) [15]

Criterion	Description
**Affordability**	Interventions often have an implicit or explicit budget. It does not matter how effective, or even cost-effective it may be if it cannot be afforded. An intervention is affordable if within an acceptable budget it can be delivered to, or accessed by, all those for whom it would be relevant or of benefit.
**Practicability**	An intervention is practicable to the extent that it can be delivered as designed through the means intended to the target population. For example, an intervention may be effective when delivered by highly selected and trained staff and extensive resources but in routine clinical practice this may not be achievable.
**Effectiveness and cost-effectiveness**	Effectiveness refers to the effect size of the intervention in relation to the desired objectives in a real world context. It is distinct from efficacy which refers to the effect size of the intervention when delivered under optimal conditions in comparative evaluations. Cost-effectiveness refers to the ratio of effect (in a way that has to be defined, and taking account of differences in timescale between intervention delivery and intervention effect) to cost. If two interventions are equally effective then clearly the most cost-effective should be chosen. If one is more effective but less cost-effective than another, other issues such as affordability, come to the forefront of the decision making process.
**Acceptability**	Acceptability refers to the extent to which an intervention is judged to be appropriate by relevant stakeholders (public, professional and political). Acceptability may differ for different stakeholders. For example, the general public may favour an intervention that restricts marketing of alcohol or tobacco but politicians considering legislation on this may take a different view. Interventions that appear to limit agency on the part of the target group are often only considered acceptable for more serious problems (Nuffield Council on Bioethics, 2007).
**Side-effects/safety**	An intervention may be effective and practicable, but have unwanted side-effects or unintended consequences. These need to be considered when deciding whether or not to proceed.
**Equity**	An important consideration is the extent to which an intervention may reduce or increase the disparities in standard of living, wellbeing or health between different sectors of society.

### Study context

In 2019, the UK National Institute for Health Research (NIHR) reinvested in 15 Applied Research Collaborations (ARCs) to tackle some of the most pressing health and social care issues in England. In October 2020, it launched a national priority call focused on seven areas of health: 1) Prevention, 2) Health and care inequalities, 3) Mental health, 4) Multimorbidity, 5) Adult social care and social work, 6) Healthy ageing and 7) Children’s health and maternity [18]. This call allocated a lead ARC to each priority call and asked ARCs to work collaboratively to identify and prioritise evidence-based interventions or service models for wider implementation to affect the health and social care issues for those with the greatest burden. Proposals could be submitted to more than one priority call and the scope for innovations was broad. The key remit of this call, and unlike other priority setting processes, was for the interventions/service models to have funding in place for wider implementation with additional funding provided to ARCs to research and evaluate the implementation in a 3 year programme of work (2020-2023). NIHR did not specify a prioritisation process/method and the period for selecting interventions for implementation was 6 months. The focus of this paper is on the Child and Maternity health priority call.

Stakeholder and Public and Patient Involvement and Engagement (PPIE)^1^ is established as an important aspect for priority setting health needs [19] and is a key aim of the NIHR ARC prioritisation process. There is limited information however, on how to best involve stakeholders with no one method meeting all our requirements in the priority setting process and limited evaluation on how successful engagement is, with time and funding limits highlighted as barriers [5, 17, 19]. Within the context of the CH&M programme, which has a strong PPIE ethos, we sought to engage with multiple stakeholders, who had an interest in the priority setting outcomes. This included public representatives,^2^ clinicians, practitioners and researchers. Each of these stakeholders potentially has a different set of priorities and therefore it was important to design a process that would engage, accommodate and balance their different perspectives. Neither the CHNRI nor JLA approach fitted with the remit of the CH&M priority programme as we were interested in evidence-based interventions/service models that meet a balance of requirements. We therefore designed a process, using the APEASE criteria, which would fit the timeframe and remit of the CH&M priority programme; this flexible approach was needed to meet the challenges of conducting this process online during the Covid-19 pandemic The aim of this paper is to describe and present the results of the process and critically evaluate the use of this approach so others may learn from our experience.

## Methods

**Fig. 1:**
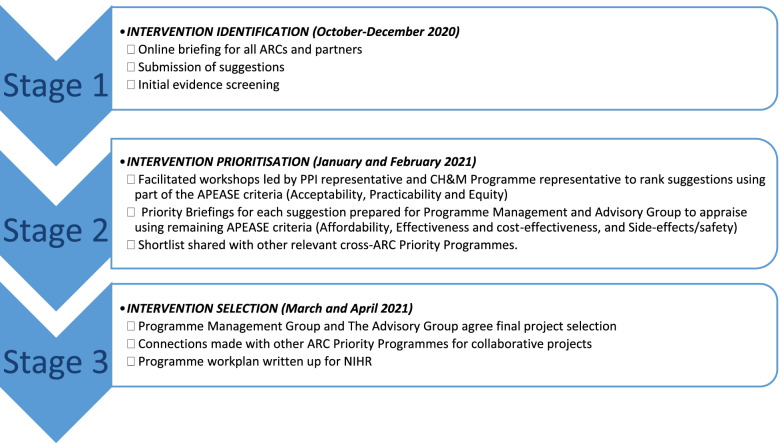
Child Health and Maternity prioritisation process

This prioritisation process took place during the Covid-19 pandemic and therefore the methods accommodated an on-line platform. A three-stage process facilitated the identification and prioritisation of evidence-based interventions or service models (Fig. 1). Stage 1 was a crowdsourcing activity primarily to identify evidence-based interventions or service models. We contacted relevant stakeholders and partners through our networks of providers, commissioners, charities and ARCs to identify interventions. We also contacted PPIE leads in the collaborating ARCs to establish networks, introduce the programme of work and disseminate the call. We hosted an on-line briefing session to encourage, support proposals, and explain the remit. We set up a programme website [20] with up-to-date information and a system for submitting proposals. Each proposal completed a simple on-line pro forma (Additional files 1 and 2); this was developed to capture basic information of the submitter and the proposed intervention/service for wider implementation, any known detail about the intervention was captured using the APEASE criteria as a framework. We offered support to complete the pro forma to all submitters if needed. After receiving the proposals, we conducted an initial review of the evidence, based on references provided by the submitter or a scan of the available evidence if none were provided.

Stage 2 focused on prioritisation using the APEASE criteria. We divided the criteria into two parts to suit the next two stages of the process. We wrote a one-page summary, in plain English, for each short-listed proposal. Where any technical or topic-specific terms were necessary in the summaries, we added these to an accompanying glossary. If there was missing information, we contacted the submitter. Due to having a wide variety of stakeholders to involve, we conducted four separate, themed on-line workshops; themes were based on the overarching topic areas of the short-listed proposals. The workshops were chaired by a public collaborator^3^ and supported by a member of the CH&M Programme Management Group (PMG) and programme staff.

At each workshop, we asked the collaborating ARCs to invite three relevant participants (one researcher, one clinician/practitioner and one public representative) from their network to ensure an even distribution of interests. For the workshops, we considered three of the APEASE criteria (Acceptability, Practicability and Equity) as these were most relevant to the expertise and experience of stakeholders attending, were considered critical to informing a comparison about readiness for implementation, and could be covered in the time available. Prior to the workshops, participants received document packs containing a proposal summary, glossary of terms, and explanations of the three relevant APEASE criteria. Public representatives also received contact details of the programme’s PPIE coordinator and details of two online support sessions that were held the week prior to the workshops. The support sessions were open discussions around topics that attendees felt needed clarification, such as the format of the workshop, where it fits within the programme, its agenda, attendees and their roles, and the APEASE criteria and scoring system. During the workshops someone from the submitting team presented their proposal in a five-minute slot framed around the 3 criteria with 10 min to answer follow-up questions from participants; the presenters then left the workshop. The participants had a general discussion about the proposals; speakers declared any conflicts of interest. Participants gave each proposal an overall score (scale range = 1-10) against the three criteria using an anonymous online poll built into the videoconferencing software. This scoring system resulted in a shortlist of the nine most highly scoring proposals across all four workshops.

We circulated a short survey (Additional file 4) after the workshops to capture stakeholders’, researchers’, clinicians’/practitioners’ and public representatives’ views of participating and their experience of the process. The survey contained five questions concerning the organisation, preparation and conduct of the workshops, as well as the participants’ perceived impact. Answers were collected by a mixture of multiple-choice checkboxes and open-ended entries. Additionally, the programme’s PPIE coordinator met online with the workshop chairs to provide a forum for feedback and discussion around the processes and support in stage 2. This was to collect information regarding the participants to help us assess the impact of the involvement process, generate learning and drive improvement.

Stage 3 concentrated on selecting 3-4 projects, from the stage 2 short-list. Priority Briefings were prepared for each of the nine short-listed proposals to evaluate the evidence in more depth, consider alignment with national health agendas and consider three further APEASE criteria (Effectiveness and cost-effectiveness, and Side-effects/safety). For the Priority Briefings, we created evidence search strategies, rapidly reviewed the top-ranking evidence and wrote a summary in plain English. The PMG (made up of ten representatives from the ARC collaborations and two public contributors) met, online, to discuss and rank the final proposals using the priority briefings and comments from the workshops. The ranking system involved all members anonymously selecting their first, second and third choices in an online poll, which resulted in a ranked list of proposals. The top three ranked proposals were removed from the list and members ranked their top three proposals from the remaining six. This process produced a set of top three ranked proposals and a reserve list of three proposals to consider if there were sufficient resources to adopt additional projects. Members declared any conflicts of interest. They also considered the final aspect of the APEASE criteria, Affordability, by assessing the immediate costs of each project in terms of committed funds for delivery and having commissioners as partners. The external Advisory Board (made up of nine external representatives from the Royal Colleges of Nursing, Obstetrics and Gynaecology, and Paediatrics, National Health Service Specialty Advisors, and other national health service leaders, and four PPIE members) sense-checked the PMG ranking against existing national priorities. We shared the short-listed proposals with collaborating ARCs and other cross-ARC priority programmes to identify any cross over projects.

The REPRISE guideline was used to report the methods of this priority setting process [21].

## Results

**Fig. 2:**
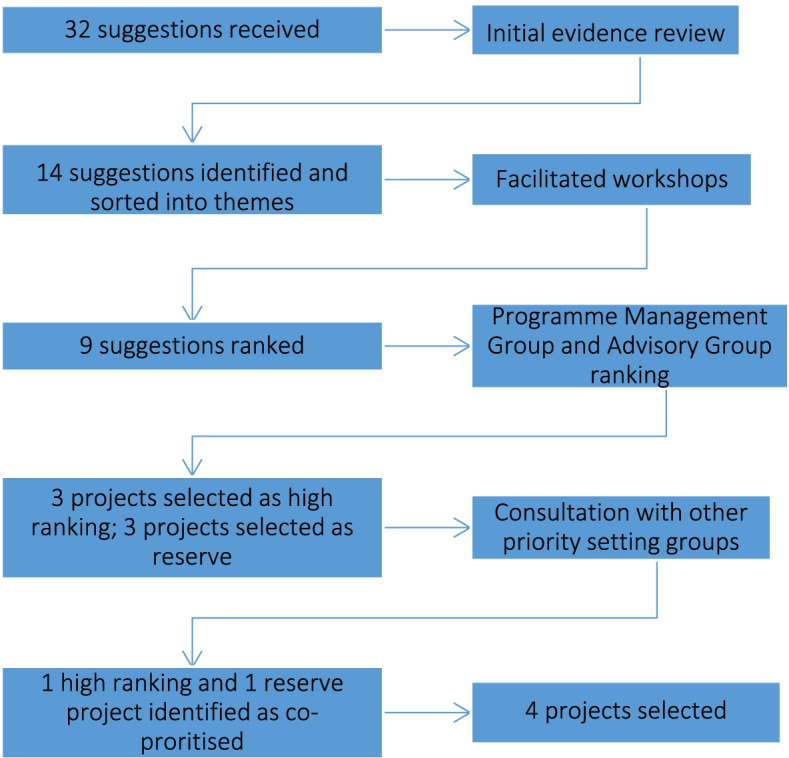
Results of Child Health and Maternity programme prioritisation

Figure 2 illustrates the results of the prioritisation process. Stage1: 86 participants attended the online briefing. After the initial call we received 32 proposals (Additional file 3), evenly split between child and maternity health. The initial review of the evidence eliminated 18 of the proposals because they were not sufficiently developed for this programme of work.

Stage 2: We sorted the 14 short-listed proposals into four themed online workshops (two maternity and two child health): 1) Antenatal care and maternity services; 2) Child mental health and Public Health; 3) Childhood disability and vulnerable populations; 4) Childbirth and maternal mental health. Table 2 indicates how each of the 14 short-listed proposals met the APEASE criteria, results of the workshop poll and Programme Management Group ranking.

**Table 2:** Results of the prioritisation process

	Suggestion	Affordability	Practicability	Effectiveness and cost-effectiveness	Acceptability	Side-effects/safety	Equity	Workshop Poll ranking in order	PMG priority rankings in order
1.	Transition of young people with long-term conditions from children to adults’ services	✓	✓	x	✓	✓	✓	7	low
2.	Early years tooth brushing programme (3-5 yr olds)	✓	✓	✓	✓	✓	✓	5	2
3.	Trauma-focused Cognitive Behaviour Therapy (TF-CBT) for children in care	✓	✓	✓	✓	✓	✓	4	3
4.	SLEEPIO	?	✓	?	✓	✓	✓	7	low
5.	The Daily Mile	?	✓	?	?	✓	✓	3	low
6.	Birmingham Symptom Specific Obstetric Triage System (BSOTS)	✓	✓	?	✓	✓	✓	6	5
7.	PERIPrem	?	✓	?	✓	✓	✓	2	6
8.	Maternal Mental Health Services Multidisciplinary Teams	✓	✓	✓	✓	✓	✓	1	1
9.	Independent Domestic Violence Advisors (IDVAs) in maternity settings	✓	✓	✓	✓	✓	✓	7	4
10.	Hospital Communications	✓	✓	x	✓	✓	✓	12	n/a
11.	Remote antenatal care for women with and without hypertension	?	✓	x	✓	✓	✓	11	n/a
12.	Probiotics as part of a Necrotizing Enterocolitis Care bundle in high-risk preterm babies	?	✓	?	✓	?	✓	9	n/a
13.	Continuity of Care for BAME pregnant women and those in deprived areas	?	✓	?	✓	✓	✓	8	n/a
14.	New Global World Health Organisation Labour Care Guide intervention	✓	✓	x	✓	✓	✓	10	n/a

‘✓’ met the criteria; ‘?’ it was not clear if the criteria was met; ‘x’ criteria was not met; *PMG* Programme Management Group

Workshop 1 had 25 participants made up of eight clinicians/practitioners, eight public representatives and nine researchers.

Workshop 2 had 24 participants made up of five clinicians/practitioners, ten public representatives and nine researchers.

Workshop 3 had 22 participants made up of three clinicians/practitioners, eight public representatives and eleven researchers.

Workshop 4 had 24 participants made up of eight clinicians/practitioners, eight public representatives and eight researchers.

See Additional file 5 for details of clinicians/practitioner’s roles. Each workshop considered 3-4 proposals. This process produced six high-ranking proposals, three medium ranking and five low ranking.

Stage 3: We prepared nine priority briefings for the high and medium ranking proposals (Additional file 1). The PMG ranked three high ranking and three reserve projects. The high-ranking three (listed in order of ranking):

1. Maternal Mental Health Services Multidisciplinary Teams
2. Early years tooth brushing programme
3. Trauma-focused CBT for young people in care

The three in reserve (listed in order of ranking):

1. Independent Domestic Violence Advisors in maternity settings
2. Birmingham Symptom Specific Obstetric Triage System (BSOTS)
3. PERIPrem

The external Advisory Board reviewed these six. We held three projects in reserve because negotiations with submitting teams had not taken place and we wanted to ensure that proposals had sufficient funding and staff available to enable implementation. We were also aware that some proposals had been submitted to and prioritised by other priority programmes within the national ARC programmes of work. This was the case for two of our highest ranked proposals (Trauma-focused CBT for young people in care and Independent Domestic Violence Advisors in maternity settings) which were also prioritised within the Health and Care Inequalities and Prevention priority themes. Negotiations therefore took place to achieve cross-programme working so that we could adopt both of these projects and prioritise four projects in total.

Sixteen out of the 84 participants responded to the post-workshop survey; 10 of those were PPIE contributors, three researchers, two clinicians and one project presenter. Overall, responders felt that the workshops were well organised and supported, although the response rate was low. The document pack they had received in advance was helpful in providing context to the process and support in preparing for the session. Most responders felt there was clear guidance on the process and their role within it. In addition, public members expressed that the sessions were interesting, well conducted and that they had enough opportunity to express their opinion.

*“Yes, I did enjoy the process probably because it was well organised, and I got the information I needed before hand and afterwards.”* (PPIE representative)

The workshop chairs also noted that the sessions flowed well, and the planning, material and support given before, after and during the sessions put them at ease. They were happy to be involved in something they are passionate about because of their own lived experiences, and found the process and projects interesting. Additionally, they emphasised that it was a great opportunity to connect with different people from all over the country.

*“I think it was great to be involved in the workshop because I am passionate about maternity service. […] I feel privileged to have chaired the meeting cause we all did such a great job”* (Workshop chair)

Some public members noted that it would have been beneficial to provide more time to discuss the individual proposals with the other panel members to come to a decision around scoring. They suggested that future events should plan for longer open discussions or breakout rooms after the presentations to continue the conversation.

*“I enjoyed the process, but I felt maybe if were given more time to discuss with other members […] we could have been more sure about our choices.”* (PPIE representative)

Two public representatives attended each of the PPIE support sessions held prior to the workshops. There was a strong sense that public representatives attending the support session were unsure how their knowledge/experience as a service user would fit into a mixed panel of professionals and sought clarification on their role within these panels. For example, attendees wanted to know whether their opinions and voting would be given equal weight and whether public representatives were represented in the workshops in equal numbers to the professionals. Attendees also appeared concerned about the level of detail in the scoring system and wondered how their personal experiences could be applied to the APEASE criteria so that they could make a well-informed decision. To address this concern, the criteria was discussed and applied to study examples to elicit questions they may want to think about during the workshop, to deduce if a proposal may or may not be worth pursuing from the perspective of their context and experiences as service users. For example: 1) is the project important to me and/or my community? 2) Is the project taking diverse communities and their needs into account? 3) Are the methods practical for me and/or my community? 4) Will the intervention reduce the burden of my community?

## Discussion

This paper outlines and reflects on the priority setting process that the CH&M priority programme designed to prioritise evidence-based interventions for implementation research projects during the Covid-19 pandemic. The need to involve a diverse group of stakeholders strongly influenced the design of this process and the specific use of the APEASE criteria in this process is unique. The results demonstrate the feasibility of the approach we took, however we acknowledge that few of the stakeholders responded to the post-workshop feedback survey and response bias may be influencing our conclusions.

There were a number of challenges to this priority setting process which took place during the Covid-19 pandemic and needed to be flexible to an online platform. Firstly, combining child health and maternity services into one priority theme with no parameters set for specific populations, problems, or settings limited the possible number of projects that the programme could adopt in either area; attention to ensure that both had equal opportunity was necessary. Secondly, the national focus is similar to priority setting in health generally, however it might exclude smaller, regional projects, which have strong local traction. There could be benefits of priority setting regionally where proposals meet the contextual needs of that locality. Arguably, however, a national focus allows for consistency of care and avoids a ‘postcode lottery’ of access to services. Thirdly, the research timeline of three years for this programme to deliver projects and achieve impact meant the deliverability of proposals needed careful consideration. Fourthly, there was a short time-line for proposals to be submitted which given we were in the winter months of a pandemic might have meant clinicians/practitioners were less able to contribute. Finally, we did not come across a process or framework that suited our CH&M priority programme and therefore designing a process and selecting methods that fitted with the needs of our stakeholders was required. A potential consequence of directing this call for evidence-based interventions that are ready and have funding for implementation could mean that projects that are less developed miss out on possible development and roll-out and thus making sure that these proposals are not lost is important.

The process we designed differs from other priority setting processes and reflects a few contextual concerns. Firstly, this process took place during the Covid-19 pandemic and therefore we needed a method that was flexible and would work in an online platform. There are advantages and disadvantages to this format, for instance it can mean stakeholders find it easier to attend, however conversely the online platform can change the nature of the exchange compared with face to face and make it harder for participants to engage and offer their views [22]. Our experience is that online meetings, when used effectively, can facilitate inclusion and opportunities for equal voice via strong chairing (limiting dominant voices and encouraging silent ones) and the use of additional technology, such as the ‘chat’ function and anonymous ranking/scoring, for those who find voicing perspectives difficult. Secondly, we wanted to ensure we had a balanced voice from our stakeholders; hence, we limited the number and type of participants invited to the workshops. As a third of the attendees were public representatives, we took advice from in-house PPIE staff (employed to facilitate and support public collaboration) on how to enable proactive contributions rather than passive responses, from public representatives. We were also aware that some submitters were non-researchers and might need support in preparing their proposal, which is why we hosted a briefing session and offered support if needed. Finally, we were aware of the need to limit the time of the online workshops for people’s comfort; hence, we split the APEASE criteria into two so that the stakeholders who attended the workshops only needed to consider the criteria that was relevant to their expertise.

The strengths of this process were its flexible approach, the resourcing of research, support and PPIE staff, being part of a national ARC network, regular team meetings with good and open communication and transparent methods. This process was, however, not without its limitations. A limitation of crowdsourcing intervention ideas is that we do not know to what extent we captured all relevant, scalable, evidence-based CH&M interventions/service models. However, a benefit of this approach is that we have captured the ideas that had support from providers/commissioners since this was a criterion for recommendation. A further limitation was that despite the aspiration to design a process that would encourage impartiality and objectivity, stakeholders inevitably have their own agendas, be it research interests, passions or particular perspectives. Notwithstanding the APEASE criteria working well, we reflected that it failed to capture the emotion or advocacy that stakeholders felt about the proposals and the potential for that to influence the prioritisation. It could be argued that adding a small ‘e’ for emotion after acceptability, A(e)PEASE, would acknowledge that it is not always possible to be objective in priority setting. Although stakeholders, PMG and Advisory Board members declared any conflicts of interest, this did not mean they ranked the proposals objectively. There is often a tension within stakeholder initiatives between objectivity and passion; however, we designed a transparent process with explicit criteria to allow for challenge if needed. Previous research acknowledges the missing component of emotion in policy deliberation in public health systems from participants with lived experience, and suggests encouraging techniques that support ‘emotional literacy’ in the process [23, 24], however researchers and clinicians equally advocate for their areas of work. Arguably, this is not something that future processes can or even should avoid, but recognising it as part of the process and criteria could be important.

## Conclusion

The priority setting process designed to select 3-4 projects in the CH&M programme during the Covid-19 pandemic achieved its aim. We had a well-balanced voice from our stakeholders and supported their involvement throughout the process. The use of APEASE criteria as an evaluative tool in the priority setting process was a novel, flexible approach that worked with the methods we selected and could be applicable to other priority setting programmes.

## Supplementary Information

**Additional file 1.**

**Additional file 2.**

**Additional file 3.**

**Additional file 4.**

**Additional file 5.**

## Data Availability

Data sharing is not applicable to this article as no datasets were generated or analysed during the current study.

## Footnotes

1 PPIE – Public involvement is research carried out ‘with’ or ‘by’ members of the public, rather than ‘to’, ‘about’ or ‘for’ them. Public engagement encompasses the various ways research is shared with the public, and encourages researchers to listen and interact with the public. (https://www.spcr.nihr.ac.uk/PPI/what-is-patient-and-public-involvement-and-engagement)

2 Public Representatives are people identifying as patients or service users of child and maternity health services

3 Public Collaborator are patients or service users who act as partners with the research team
