# Supplementary material for: Prioritising child health and maternity evidence-based interventions or service models: a stakeholder-driven process
Source: BMC Health Serv Res. 2022 Jun 10;22:764. doi: 10.1186/s12913-022-08110-2 (PMC9186012; doi:10.1186/s12913-022-08110-2)
Supplement: Supplementary file 1 — Additional file 1. [file 12913_2022_8110_MOESM1_ESM.docx]

**Child Health and Maternity National Priority Programme intervention online form – for clinicians, commissioners, researchers and groups.**

1. Name:
2. Email:
3. Are you primarily a:
    Service user or interested member of the public
   Clinician, practitioner or service provider
    Commissioner or policy-maker
    Researcher
    Group
4. Where are you based?
    East of England
    East Midlands
    Greater Manchester
    Kent, Surrey and Sussex
    North East and North Cumbria
    North Thames
    Northwest London
    North West Coast
    Oxford and Thames Valley
    South London
    South West Peninsula
    Wessex
    West
    West Midlands
    Yorkshire and Humber
5. What area of child or maternal health are you hoping to see prioritised?
6. What Child Health or Maternity intervention/service are you suggesting for wider implementation?

1. What is the intervention/service trying to improve and for whom?
2. Do you know whether there is any evidence that the intervention/service works?
    Yes
    No
    Not sure
3. If YES, please give details (ie reference, publication, report)
4. Do you know if there are any known side-effects or safety issues to think about with this intervention/service?
    Yes

No

Not sure

1. If YES, please give details.

1. Do you know where this intervention/service has been delivered to date?
    Yes
    No
    Not sure
2. If YES, please give details.
3. Do you know people in other regions who want to deliver or commission this intervention/service?
    Yes
    No
    Not sure
4. If YES, who and in which areas?
5. Do you know people or groups using the intervention/service it is intended to serve?
    Yes
    No
    Not sure
6. If YES, how have you engaged with them?
7. Do you know if there is any funding or potential funding to deliver this intervention/service?
    Yes
    No
    Not sure
8. If YES, please give details.
