# Supplementary material for: Prioritising child health and maternity evidence-based interventions or service models: a stakeholder-driven process
Source: BMC Health Serv Res. 2022 Jun 10;22:764. doi: 10.1186/s12913-022-08110-2 (PMC9186012; doi:10.1186/s12913-022-08110-2)
Supplement: Supplementary file 2 — Additional file 2. [file 12913_2022_8110_MOESM2_ESM.docx]

**Child Health and Maternity National Priority Programme intervention online form – for service users**

1. Name:
2. Email:
3. Are you primarily a:
    Service user or interested member of the public
    Clinician, practitioner or service provider
    Commissioner or policy-maker
    Researcher
    Group
4. Where are you located (town/area)?
5. Do you know which area of child or maternal health are you hoping to see prioritised (ie maternal health, mental health)?
6. If YES, please give further details
7. What child health or maternity intervention/service are you suggesting for wider implementation?
8. What is the intervention/service trying to improve and for whom?
9. What does this intervention/service do well?
10. Where is the intervention/service provided?
11. Do you know if this intervention/service is provided elsewhere?
     Yes
     No
     Not sure
12. Do you know other people or groups who use this intervention/service?
     Yes
     No
     Not sure
13. If YES, please give further details
14. Do you know if there is any funding or potential funding to deliver this intervention/service?  Yes
     No
     Not sure
15. If YES, please give details.
