# Supplementary material for: Prioritising child health and maternity evidence-based interventions or service models: a stakeholder-driven process
Source: BMC Health Serv Res. 2022 Jun 10;22:764. doi: 10.1186/s12913-022-08110-2 (PMC9186012; doi:10.1186/s12913-022-08110-2)
Supplement: Supplementary file 3 — Additional file 3. [file 12913_2022_8110_MOESM3_ESM.docx]

Additional file 3: 32 Original suggestions

| **Suggestions short-listed for the workshops (shaded suggestions are the nine priority briefings)** | |
| --- | --- |
| 1. The Parent Infant Interaction Observation Scale (PIIOS) | A scale to aid practitioners in the identification of interactional patterns between parents and infants that could raise concern about the baby’s social and emotional development. |
| 1. Transition of young people with long-term conditions from children to adults’ services | An intervention consisting of key components that would improve transitional care for young people with long-term conditions (such as diabetes, cerebral palsy, or autism) from children to adult services. |
| 1. Hospital Communications | A training package to help hospital staff to improve communication with disabled children and their families. |
| 1. Early years tooth brushing programme (3-5yr olds) | Supervised tooth brushing with fluoride toothpaste in early years settings and schools for 3-5 year olds, focused on areas of higher deprivation. |
| 1. Trauma-focused CBT (TF-CBT) | To support the mental health of young people in care by improving current practice. |
| 1. SLEEPIO | Online sleep intervention for young people with mental health problems using CBTi. |
| 1. The Daily Mile | Whole school, teacher-led intervention, which involves every child running/jogging for 15 minutes at least three times a week. |
| 1. Probiotics as part of a Necrotizing Enterocolitis Care bundle | Use of enteral probiotics as part of a NEC Care bundle to reduce mortality, morbidity and reduction in length of stay on Neonatal Units associated with Necrotizing Enterocolitis (NEC) in high-risk Preterm babies. |
| 1. New Global WHO Labour Care Guide intervention | Positive childbirth experience because of the longer-term impact on effective mothering, parenting, and parental, family, and child wellbeing, for all mothers and babies, and especially those who are most marginalised. |
| 1. Birmingham Symptom Specific Obstetric Triage System (BSOTS) | An assessment and clinical prioritisation of the common conditions that women present with in maternity triage and a standardised way of determining the clinical urgency in which patients are seen. |
| 1. PERIPrem | Implementation of a bundle of 10 evidence-based elements including the PreCept magnesium sulphate intervention to prevent neuro-disability in preterm infants. |
| 1. Maternal Mental Health Services Multidisciplinary Teams | Evaluation of the Implementation and scale-up of Maternal Mental Health Services (multidisciplinary team) - specialist community-based assessment and intervention for women with mental ill health in maternity, neonatal and perinatal contexts. |
| 1. Continuity of Care for BAME pregnant women and those in deprived areas | Implementation and scale up of Continuity of Care (CoC) for BAME pregnant women and women living in disadvantaged areas across the country. Continuity of the midwife looking after women in maternity services. |
| 1. Independent Domestic Violence Advisors (IDVAs) in maternity settings | The aim of IDVAs is to secure the safety of those at risk of harm from intimate partners, ex-partners or family members. They work with those affected to assess risk, develop safety plans, reduce abuse and ultimately improve health and wellbeing in women and children. |
| **Suggestions not shortlisted for workshops** | |
| 1. Watch Me Play | An intervention for nursery nurses and health visitors to deliver to parents and their infants or young children to support child development and parent-infant relationships. |
| 1. Early Powered Mobility for Young Children (<5 years) with mobility limitations | Powered mobility devices such as an electric wheelchair or starter devices, environmental adaptations and technical maintenance for young children (<5 years) with mobility limitations. |
| 1. Health Visitors for Children with SEN | Designated Health Visiting service for parents of SEN children. |
| 1. Community Thrive in Action | Training for ‘trusted messengers’ (community members and volunteers in contact with families likely to experience vulnerabilities) to deliver information about brain development to parents. |
| 1. Integrated Care Team Model for families with mental health issues | A multi-discipline team with an integrated approach to support families with mental health issues stemming from deprivation through early identification and intervention. |
| 1. Promotion of healthy weight in the early years | Online weight-related training resource for frontline practitioners working with pre-school children. |
| 1. DfE's Holiday Activity and Food Programme | Delivery of the government funded school holiday programmes located in areas of multiple deprivation. |
| 1. Remote antenatal care for women with and without hypertension | Self-monitoring of blood pressure in the antenatal period. |
| 1. The Hearts Milk Bank (HMB) Lactation support intervention | Support for new mothers facing breastfeeding challenges using lactation consultants, and with access to donor human milk if supplementation is required as part of a feeding plan. |
| 1. SNUG Neonatal Pathways Project | Peer Specialist support to reduce the stress, anxiety and social isolation of families who have sick and vulnerable babies in hospital neonatal care and who require ongoing support after leaving hospital. |
| 1. Nest @Home Intervention | A co-produced service intervention that offers a family integrated approach and personalized home support package to enable late preterm babies to transfer home safely earlier. |
| 1. Prevention of Preterm Birth Intervention | Vaginal progesterone for women with singleton pregnancy at risk of preterm birth (either short cervix or previous preterm birth). |
| 1. Birth ball | A birth ball and infomercial demonstrating how to use the birth ball at home to support women to labour at home during the latent phase of labour. |
| 1. The Mums' Comfort Zone Programme | A 12-week community-based group programme for mothers experiencing perinatal mental health difficulties, alongside a community based perinatal mental-health peer support service. |
| 1. Butterfly cot cards intervention | Cards used to signify to staff that the baby in the NICU cot is a surviving baby from a multiple pregnancy. |
| 1. eHealth + mHealth | For use to improve mental health/postnatal care of poorer women in childbirth in marginalised communities. |
| 1. Graded Care Profile 2 Antenatal (GCP2-A) | An assessment tool that helps midwives, social workers and other professionals working with pregnant mothers to identify issues, which can have long-term detrimental impact on a developing baby. |
| 1. Benchmarking maternity outcomes | Each National Clinical Commissioning Group would work with maternity providers to provide data on key 10 maternity outcomes for their lowest IMD decile. |
