# Supplementary material for: Prioritising child health and maternity evidence-based interventions or service models: a stakeholder-driven process
Source: BMC Health Serv Res. 2022 Jun 10;22:764. doi: 10.1186/s12913-022-08110-2 (PMC9186012; doi:10.1186/s12913-022-08110-2)
Supplement: Supplementary file 4 — Additional file 4. [file 12913_2022_8110_MOESM4_ESM.docx]

# Children’s Health and Maternity Programme

# Prioritisation Panel feedback form

1. Did you feel the meeting was well organised by the programme team?

- Yes
- No
- Neither ‘Yes’ or ‘No’

Comments:

1. Was the document pack you received in preparation helpful?

- Yes
- No
- Neither ‘Yes’ or ‘No’

Comments:

1. Did you have clear guidance of what would happen on the day and what your role would be?

- Yes
- No
- Neither ‘Yes’ or ‘No’

Comments:

1. Did you have enough opportunity to contribute to the meeting? E.g. were you able to put your point across?

- Yes
- No
- Neither ‘Yes’ or ‘No’

Comments:

1. Did you enjoy the process? What could we have done better/differently to support the process as a whole?

Are you a:

- Service user / Public contributor
- Clinician / practitioner
- Academic
- Project presenter

Are you involved in any of the projects that were under discussion?

- Yes
- No
- Not sure

If you would like to be kept up to date about the Children’s Health and Maternity programme and/or would like to be informed about future involvement opportunities, please indicate below:

- Yes, keep me up to date about the programme
- Yes, I am interested in future involvement opportunities

# Many thanks for your feedback and support to the Children’s Health and Maternity programme.
