# Supplementary material for: Prioritising child health and maternity evidence-based interventions or service models: a stakeholder-driven process
Source: BMC Health Serv Res. 2022 Jun 10;22:764. doi: 10.1186/s12913-022-08110-2 (PMC9186012; doi:10.1186/s12913-022-08110-2)
Supplement: Supplementary file 5 — Additional file 5. [file 12913_2022_8110_MOESM5_ESM.docx]

| **Workshop 1-** *Antenatal care and maternity services; n=25* | **Workshop 2-** *Child mental health and Public Health; n=24* | **Workshop 3 -** *Childhood disability and vulnerable populations; n=22* | **Workshop 4 -** *Childbirth and maternal mental health; n=24* |
| --- | --- | --- | --- |
| *Clinical roles:* | *Clinical roles:* | *Clinical roles:* | *Clinical roles:* |
| Senior Sonographer | Clinical Director of Children’s Hospital | Transition Clinical Lead/ Paediatric Palliative Care Consultant | Service Manager |
| Consultant Paediatrician | Clinical Psychologist | Paediatric Doctor/Academic Clinical Fellow | Clinical Network Manager |
| Neonatologist x3 | Clinical Epidemiologist | Midwife | Consultant Psychiatrist |
| Midwife x2 | Consultant Public Health | Nurse | Midwife x2 |
| Consultant in Obstetrics and Fetal Medicine | Public Health Specialist |  | Specialist Midwife: Perinatal Mental Health and Complex Care |
|  |  |  | Consultant Obstetrician and Gynaecologist |
|  |  |  | Programme Manager Perinatal Mental Health |
| 8 x PPI representatives | 10 x PPI representatives | 8 x PPI representatives | 8 x PPI representatives |
| 9 x research representatives | 9 x research representatives | 10 x research representatives | 8 x research representatives |
